# Forced Expression of Nanog or Esrrb Preserves the ESC Status in the Absence of *Nucleostemin* Expression

**DOI:** 10.1002/stem.1918

**Published:** 2014-12-18

**Authors:** Miyuki Katano, Masatsugu Ema, Yutaka Nakachi, Yosuke Mizuno, Masataka Hirasaki, Ayumu Suzuki, Atsushi Ueda, Masazumi Nishimoto, Satoru Takahashi, Yasushi Okazaki, Akihiko Okuda

**Affiliations:** a Division of Developmental Biology, Saitama Medical UniversityYamane, Hidaka, Saitama, Japan; b Department of Anatomy and Embryology, Institute of Basic Medical Sciences, Graduate School of Comprehensive Human Sciences, University of TsukubaTsukuba, Japan; c Division of Translational Research, Saitama Medical UniversityYamane, Hidaka, Saitama, Japan; d Division of Functional Genomics and Systems Medicine, Saitama Medical UniversityYamane, Hidaka, Saitama, Japan; e Radioisotope Experimental Laboratory, Research Center for Genomic Medicine, Saitama Medical UniversityYamane, Hidaka, Saitama, Japan; f Core Research for Evolutional Science and Technology (CREST), Japan Science and Technology AgencyKawaguchi, Saitama, Japan

**Keywords:** Embryonic stem cells, Epiblast stem cells, Leukemia inhibitory factor, Pluripotency

## Abstract

Nucleostemin (NS) is a nucleolar GTP-binding protein that is involved in a plethora of functions including ribosomal biogenesis and maintenance of telomere integrity. In addition to its expression in cancerous cells, the *NS* gene is expressed in stem cells including embryonic stem cells (ESCs). Previous knockdown and knockout studies have demonstrated that NS is important to preserve the self-renewality and high expression levels of pluripotency marker genes in ESCs. Here, we found that forced expression of Nanog or Esrrb, but not other pluripotency factors, resulted in the dispensability of *NS* expression in ESCs. However, the detrimental phenotypes of ESCs associated with ablation of *NS* expression were not mitigated by forced expression of Rad51 or a nucleolar localization-defective NS mutant that counteracts the damage associated with loss of *NS* expression in other *NS*-expressing cells such as neural stem/progenitor cells. Thus, our results indicate that *NS* participates in preservation of the viability and integrity of ESCs, which is distinct from that in other *NS*-expressing cells. Stem Cells *2015;33:1089–1101*

## Introduction

Nucleostemin (NS) (also known as GNL3) is a nucleolar GTP-binding protein that is involved in numerous biological functions including ribosome biogenesis, protection against telomere damage, and destabilization of p53 protein [1–5]. Recently, a genome-protecting role of NS has been uncovered in neural stem cells (NSCs) [6] and regenerating hepatocytes [7], in which NS protein recruits Rad51 to foci of DNA damage to facilitate DNA repair. NS was first discovered in NSCs [1] and later in numerous types of stem/progenitor cells and cancer cells. Stem cells are defined by their abilities to produce multiple distinct cell types (multipotency) and self-renew while maintaining their multipotency. However, each stem cell type is usually only able to generate cell types that coexist in certain tissues [8]. For example, NSCs produce only neurons, astrocytes, and oligodendrocytes in the brain, at least in a physiological context [9]. In terms of self-renewality, the durations are different depending on the stem cell type, but it is never permanent. Embryonic stem cells (ESCs) are also categorized as stem cells, although their differentiation potential and self-renewal properties are much greater compared with those of other stem cell types, because they can convert to any cell type in the body, including germ cells, and maintain self-renewality indefinitely [10–12]. NS is also expressed in ESCs, and we have previously demonstrated such expression is essential to preserve cell viability [13]. Another study [4] has shown that *NS* expression is important for rapid transit through G1 phase to sustain the robust proliferation of ESCs and is also crucial to maintain high expression levels of pluripotency marker genes. However, the molecular mechanisms of NS in ESCs remain largely unexplored. Moreover, it is unknown whether *NS* expression is crucial for other pluripotent cells, that is, epiblast stem cells (EpiSCs) [14,15].

Here, we demonstrate that ablation of *NS* expression leads to a substantial decline in the expression levels of pluripotency marker genes and extensive cell death of both EpiSCs and ESCs. However, unlike in NSCs, the effects of *NS* expression ablation are not mitigated by forced expression of Rad51, implying a pluripotent cell-specific function of NS. Our data also demonstrate that the changes associated with *NS* expression ablation in ESCs, but not in EpiSCs, are almost completely rescued by forced expression of either Nanog or Essrb pluripotency factors.

## Materials and Methods

### Cell Culture

Wild-type (CMTI-1) and *NS* tet-off ESCs, in which *NS* gene expression from the *ROSA26* locus can be controlled by the tetracycline-off system [13], were cultured under a feeder-free condition with standard ESC medium containing leukemia inhibitory factor (LIF) and serum [16]. To generate *NS* tet-off EpiSCs, *NS* tet-off ESCs were marked with fluorescent Kusabira Orange (KBO) by stable integration of a KBO expression vector with a puromycin resistance gene [17] and then injected into blastocysts. After transfer to surrogate ICR mice, embryos at ∼6.5 days postcoitum (dpc) were recovered to establish EpiSCs according to Brons et al. [15]. The EpiSCs were cultured on fibronectin-coated dishes with medium containing 20 ng/ml human activin A (338-AC-050) (R&D Systems, Minneapolis, MN, http://www.rndsystems.com) and 12 ng/ml murine basic fibroblast growth factor (450-33) (PeproTech, Rocky Hill, NJ, http://www.peprotech.com) as described by Gillich et al. [18]. To suppress *NS* expression from the *ROSA26* locus in the inducible *NS* tet-off ESCs and EpiSCs, 1 µM doxycycline (Dox) was added to the culture medium.

### TUNEL Assay

For TUNEL assays, cells were plated on gelatin-coated Cell Disks. TUNEL-positive apoptotic cells were detected using an In situ Cell Death Detection kit, Fluorescein (11684795910) (Roche Applied Science, Mannheim, Germany, http://www.roche-applied-science.com).

### Hydroxyurea Treatment

CMTI-1 ESCs were transiently transfected with expression vectors for either wild-type or the ΔB mutant of NS [19] and then treated with 2 mM hydroxyurea (HU) for 20 hours. Subsequently, HU-containing medium was replaced by standard ESC medium and then cultured for 4 hours before subjecting to immunocytochemistry using an antibody against γ-H2A-X. CMTI-1 ESCs transfected with an empty vector were used as a control.

### Microarray Analysis

Biotin-labeled cRNA was synthesized as described by the Affymetrix guidelines. Labeled samples were hybridized to Affymetrix GeneChip Mouse Genome 430 2.0 arrays according to the manufacturer's instructions. Microarray expression data were background subtracted and normalized with the robust multiarray analysis method using statistical software R. Gene ontology (GO) analyses were conducted using DAVID web tools (http://david.abcc.ncifcrf.gov). The selected GO terms were further subjected to analyses using AmiGO1 (http://amigo1.geneontology.org/cgi-bin/amigo/go.cgi) and REVIGO (http://revigo.irb.hr) web sites to eliminate redundancy. For gene set enrichment analysis (GSEA), we used GSEA software v2.0.14.

### Quantitative RT-PCR

Quantitative RT-PCR was conducted with the StepOnePlus real-time PCR system (Applied Biosystems, Foster City, CA, http://www.appliedbiosystems.com) using cDNAs generated by reverse transcription. The TaqMan-based reactions quantified the expression levels of *Gapdh*, *Nanog*, *Oct3/4*, *Sox2*, *Sall4*, *Utf1*, *Tbx3*, *Tcl1*, *Esrrb*, *Rex1*, *Klf2*, *Klf4*, *Gata4*, *Gata6*, *T*, *Cdx2*, and *Pax6*. SYBR Green-based reactions were used to quantify the expression levels of *Prdm14* with the following primer set: forward, 5′-GGC CAT ACC AGT GCG TGT A-3′; reverse, 5′-TGC TGT CTG ATG TGT GTT CG-3′. The results were normalized to *Gapdh* expression levels.

### Alkaline Phosphatase Staining

ESCs (2,500 cells) were plated in each well of a gelatin-coated six-well plate and cultured with or without Dox. Then, the cells were stained with an AP staining kit (AP100R-1) (System Biosciences, Inc., Mountain View, CA, http://www.systembio.com).

### Knockdown of NS Expression in Human-Induced Pluripotent Stem Cells

Human-induced pluripotent stem cells (hiPSCs) (SC102A-1) were cultured according to the supplier's instructions (System Biosciences, Inc.). Inhibition of *NS* expression by shRNA-mediated knockdown in hiPSCs was conducted as described previously [20] with the sequence (shNS1) used by Okamoto et al. [21].

### Accession Number

DNA microarray data have been deposited in the NCBI Gene Expression Omnibus under accession number GSE56797.

Materials used in this study, vector constructions, Western blot, and immunostaining methods were provided in Supporting Information Materials and Methods.

## Results

### Decline in the Expression Levels of Pluripotency Marker Genes After Ablation of NS Expression in ESCs

**Figure 1:**
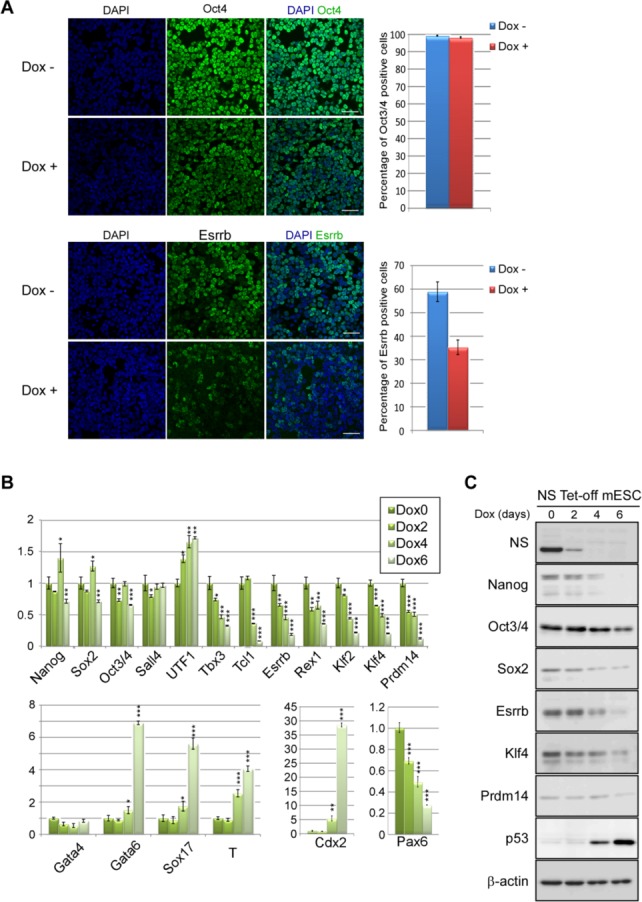
Most pluripotency marker genes undergo a decline in expression after ablation of *NS* expression in ESCs. (A): Immunocytochemical analyses of Oct3/4 and Esrrb in Dox-untreated and -treated *NS* tet-off ESCs. *NS* tet-off ESCs were cultured on cell disks in the absence or presence of Dox for 5 days before immunocytochemistry. The ratio of Oct3/4- or Esrrb-positive cells among DAPI-positive cells is shown as the mean ± SD (*n* = 3). Scale bar = 50 µm. (B): Quantitative RT-PCR analyses of pluripotency and differentiation marker expression. Data are the mean ± SD (*n* = 3). Each value from Dox-untreated *NS* tet-off ESCs is set to one. *, *p* < .05; **, *p* < .01; and ***, *p* < .001. (C): Western blot analyses of pluripotency marker and p53 proteins. *NS* tet-off ESCs were treated with Dox and recovered at the indicated days. Abbreviations: ESC, embryonic stem cell; NS, nucleostemin.

We have previously demonstrated that ablation of *NS* expression leads to extensive cell death of ESCs using inducible *NS* tet-off ESCs in which the *NS* gene was homozygously disrupted, but NS cDNA was introduced into the *ROSA26* locus together with the tetracycline-off system [13]. Furthermore, we demonstrated with that the expression levels of pluripotency markers such as Oct3/4 and UTF1 are not noticeably influenced by the loss of *NS* expression in this ESC line. However, a recent report with knockdown experiments of *NS* expression in ESCs demonstrated that a decline in *NS* expression levels does not provoke a strong apoptotic phenotype, but lowers the expression levels of numerous pluripotency marker genes [4]. We considered that these apparent discrepancies may be attributed to a difference in the levels of residual *NS* expression in *NS*-knockout and -knockdown ESCs, resulting in a milder phenotype in *NS*-knockdown ESCs with respect to cell viability. In terms of pluripotency marker gene expression, we assumed that the extensive cell death phenotype of *NS*-knockout ESCs had prevented us from correctly quantifying the expression levels of pluripotency marker genes. To address the latter possibility, we first examined Oct3/4 expression in *NS*-knockout ESCs by immunocytochemical analyses. Although our previous Western blot and mRNA quantification analyses did not indicate a decline in Oct3/4 expression after Dox administration [13], immunocytochemistry showed some differences in Dox-untreated (*NS* expressing) and -treated (*NS* nonexpressing) *NS* tet-off ESCs. Indeed, cells with a low intensity of Oct3/4 immunostaining were apparent among Dox-treated *NS* tet-off ESCs. However, most of them were not completely negative, but retained at least certain signal for Oct3/4. Therefore, the proportion of completely negative cells for Oct3/4 was similar between Dox-treated and -untreated *NS* tet-off ESCs. These results were in marked contrast to the results obtained with Dox-untreated *NS* tet-off ESCs that showed homogeneous Oct3/4 signals (Fig. 1A). We also performed immunocytochemical analyses of Esrrb. Consistent with previous reports [22–24], Esrrb expression was heterogeneous among ESCs with only approximately 60% of ESCs positive for Esrrb even among Dox-untreated *NS* tet-off ESCs (Fig. 1A). However, the proportion of cells negative for Esrrb became much more prominent following Dox treatment, implying that the overall expression level of Esrrb was also decreased upon *NS* expression ablation. Furthermore, analyses of the expression of Nanog, which is also a pluripotency marker with rather heterogeneous expression in ESCs [23], revealed that the proportion of Nanog-negative cells was increased significantly after ablation of *NS* expression in ESCs (Supporting Information Fig. S1).

Because our immunocytochemical analyses indicated downregulation of the expression of Oct3/4, Esrrb, and Nanog, we determined whether other pluripotency marker genes underwent downregulation upon Dox-mediated ablation of *NS* expression in ESCs. Quantitative RT-PCR analyses demonstrated that most of the examined pluripotency marker genes underwent significant downregulation of expression. However, some pluripotency marker genes such as *Oct3/4*, *Sox2*, and *Sall4* were not significantly influenced by the loss of *NS* expression. The *UTF1* expression level was even elevated in *NS*-null ESCs (Fig. 1B). Thus, these findings partly account for the previous discrepancy in the decline of pluripotency marker gene expression in Dox-treated *NS* tet-off ESCs [13]. Differential effects of *NS* expression ablation on gene expression among pluripotency markers were also consistent with a previous report [4]. Western blot analyses also showed decreases in the expression of all the pluripotency marker proteins, particularly Nanog, Esrrb, and Klf4, upon Dox-mediated loss of *NS* expression (Fig. 1C). Again, the decline in the expression level of Oct3/4 was not very obvious compared with that of other pluripotency markers such as Nanog and Esrrb. Thus, our data indicate that pluripotency marker genes can be categorized into two groups according to rapid or gradual declines in their expression levels (first and second groups, respectively). Therefore, a reduction in the expression levels of second group genes such as Oct3/4 may be due to a secondary effect of the reduction in the expression levels of first group genes such as Klf4 and Esrrb. Consistent with this notion, we observed a strong rescue effect by forced expression of certain first group genes (see below). With respect to the expression of differentiation marker genes, we noted induction of T (Brachyury) expression, implying initiation of mesodermal induction. However, there was no elevation in the expression levels of ectodermal (Pax6) or endodermal (Gata4) marker genes, while Gata6 and Sox17, other endodermal markers, showed increases in their expression levels upon loss of NS expression in ESCs. Unexpectedly, we found the trophectodermal marker gene Cdx2 underwent a profound increase in expression (Fig. 1B), although ESCs do not intrinsically have the ability to differentiate into the trophectodermal cell lineage. However, we do not know the reason for the abnormal expression pattern of the differentiation marker genes, especially the strong induction of Cdx2 in Dox-treated *NS* tet-off ESCs.

### Decline in the Phosphorylation Level of Stat3 in NS tet-off ESCs

**Figure 2:**
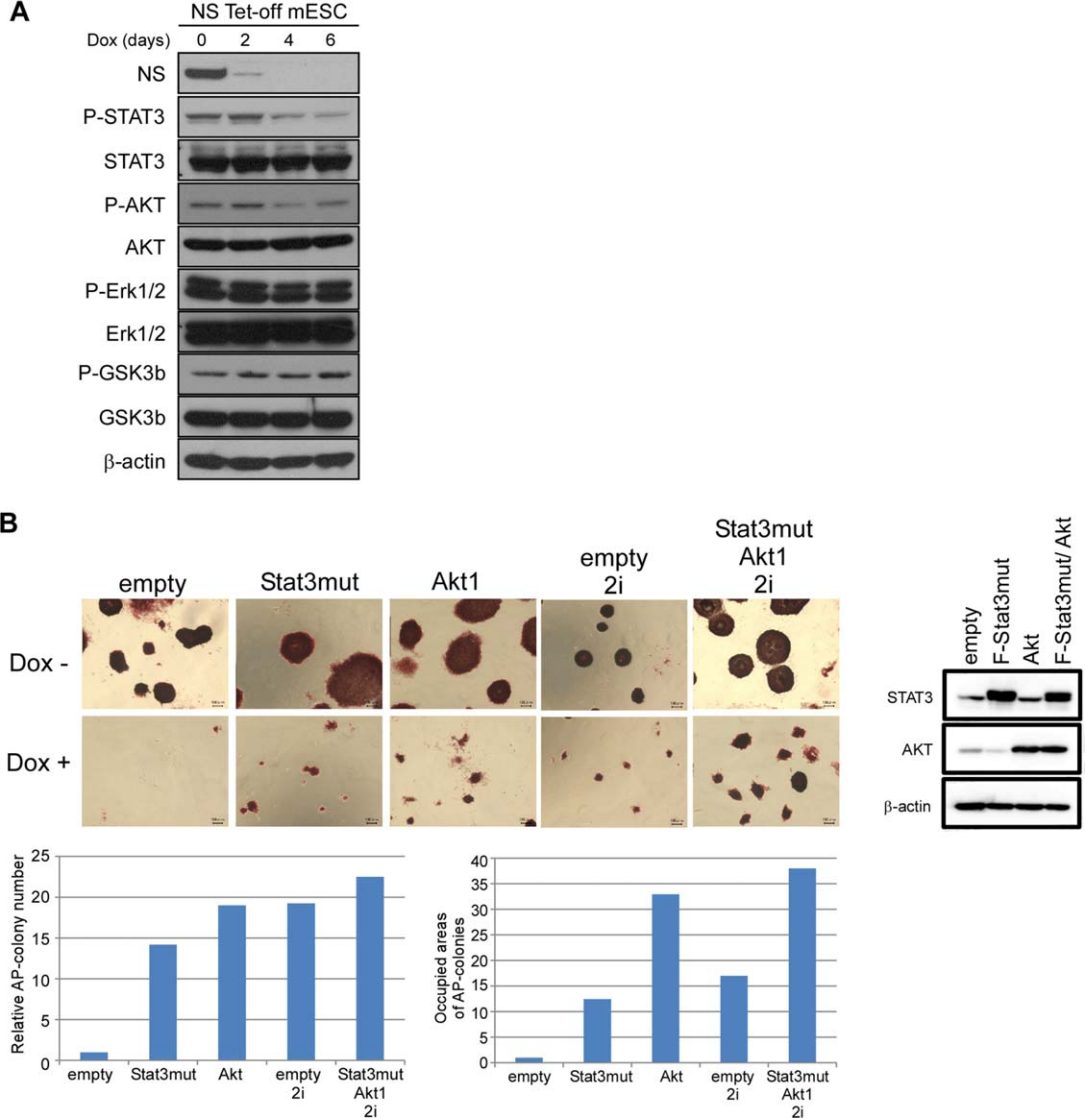
Incomplete counteraction of *NS* expression ablation-mediated detrimental phenotypes of ESCs by constitutive activation of Stat3 Akt or exposure to the 2i condition. (A): Western blot analyses to examine the phosphorylation levels of signal transduction kinases that are related to ESC pluripotency. *NS* tet-off ESCs were treated with Dox and then protein extracts were prepared at the indicated days. (B): Partial rescue of *NS* tet-off ESCs by either constitutively active Stat3, Akt, or exposure to the 2i condition. Upper right panel shows a Western blot for confirmation of the exogenously overexpressed Stat3 mutant and Akt. The combinatorial effect was also examined for all three components. Lower left and right bar graphs show the relative number of AP-positive colonies and the total occupied area of all rescued colonies, respectively, in which data from Dox-treated *NS* tet-off ESCs are set to 1 in both cases. *NS* tet-off ESCs and those overexpressing constitutively active Stat3 and/or Akt were treated as indicated for 6 days, and then subjected to AP staining. Scale bar = 100 µm. Abbreviations: ESC, embryonic stem cell; NS, nucleostemin.

**Figure 3:**
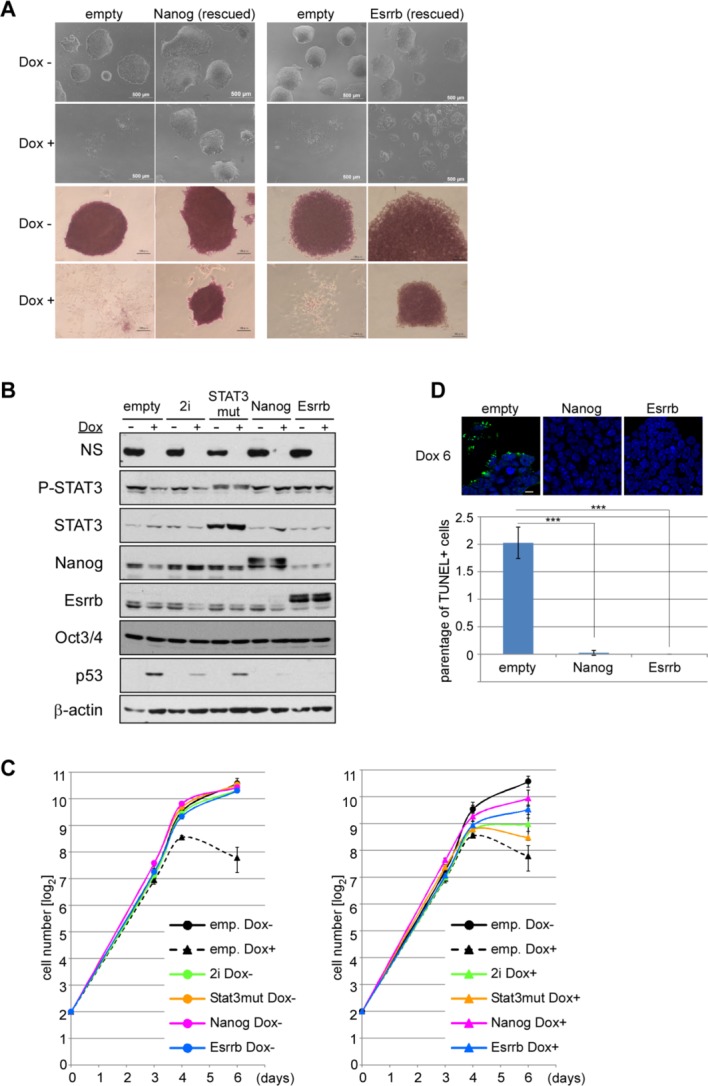
Strong rescue effects by forced expression of Nanog or Esrrb in *NS* tet-off embryonic stem cells (ESCs). (A): Forced expression of Nanog or Esrrb allowed self-renew for a prolonged period even after ablation of *NS* expression in ESCs. After stable integration of expression vectors for Nanog or Esrrb with puromycin selection, the cells were cultured in the presence or absence of Dox with standard ESC medium. After 6 days, the cells were examined under a conventional bright field microscope and then subjected to alkaline phosphatase staining. Scale bar = 500 µm (upper panel); 100 µm (lower panel). (B): Western blot analyses of cells that were partially rescued by exposure to the 2i condition or with constitutively active Stat3 and those rescued by forced expression of Nanog or Esrrb. The cells were cultured in the presence or absence of Dox for 6 days. (C): Growth curves of *NS* tet-off ESCs overexpressing either constitutively active Stat3, Nanog, or Esrrb, or exposed to the 2i condition. *NS* tet-off ESCs transfected with an empty vector were used as controls. Cell numbers were counted at the indicated days, divided by 1 × 10^4^, and then plotted on the semilogarithmic graph. The number of cells at day 0 was 4 × 10^4^. Data from Dox-untreated and -treated cells are plotted in the left and right panels, respectively. (D): TUNEL assay of Nanog- or Esrrb-overexpressing *NS* tet-off ESCs. The cells were cultured in the presence of Dox for 6 days. The ratio of TUNEL-positive cells to DAPI-positive cells is shown with the SD. ***, *p* < .001. Scale bar = 10 µm. Abbreviation: NS, nucleostemin.

To understand the molecular basis of the extensive cell death and decline in the expression levels of pluripotency genes, we explored methods to sustain the ESC status in the absence of *NS* gene expression. Pluripotency marker genes including *Klf4* and *Esrrb* show higher expression in ESCs than that in EpiSCs [25]. Furthermore, these pluripotency genes were more predominantly downregulated by *NS* expression ablation than other genes (*Oct3/4* and *UTF1*) showing apparently equivalent expression levels (Fig. 1B). Therefore, we examined the possibility that the LIF-Stat3 signaling cascade was impaired in *NS* tet-off ESCs, because ESCs, but not EpiSCs, are crucially dependent on this signaling cascade. Figure 2A shows that, although the total amount of Stat3 did not change, the amount of phosphorylated (active) Stat3 was decreased significantly upon loss of *NS* expression in ESCs. We also noted that the amount of phosphorylated Akt, which cooperatively participates with Stat3 to maintain ESC pluripotency [26,27], was also decreased in *NS* tet-off ESCs, although the decrease was less significant compared with that of phosphorylated Stat3. However, phosphorylation levels of Erk1/2 and GSK3β were unchanged by *NS* expression ablation. These results prompted us to examine the effect of forced expression of the constitutively active form of Stat3 [28] and Akt on *NS* tet-off ESCs. These analyses revealed that both constitutively active Stat3 and Akt yielded some alkaline phosphatase-positive viable colonies (Fig. 2B), although most of the cells underwent cell death in both cases. However, both Stat3- and Akt-dependent colonies showed extremely slow cell proliferation (refer Fig. 3C for Stat3; data not shown for Akt) and substantial cell death occurred during each passage. Therefore, we could not maintain either Stat3- or Akt-dependent colonies cells over several passages. The dependency of ESCs on LIF-Stat3 signaling is much weaker in ESCs under the 2i plus LIF condition compared with that in the conventional LIF and serum condition [29,30]. Moreover, previous knockdown studies have demonstrated that the decline in the expression levels of pluripotency marker genes is much less significant when *NS* expression in ESCs is knocked down under the 2i plus LIF condition [4]. Therefore, we examined the effect of *NS* knockout in ESCs under the 2i plus LIF condition. Similar to forced expression of constitutively active Stat3, we observed some alkaline phosphatase-positive viable cells (Fig. 2B), although the vast majority of *NS* tet-off ESCs underwent cell death. We assumed that the less potent effect of the 2i plus LIF condition on *NS*-knockout ESCs compared with that in *NS*-knockdown ESCs [4] was again attributed to the difference in residual *NS* expression levels in *NS*-knockout and -knockdown ESCs. Because we obtained a partial rescue effect by Stat3, Akt, or the 2i condition, we next examined the effect of the combination of all three components. The results revealed that the rescue effect was more significant when these components were applied in combination compared with the individual components in terms of both the number and size of the rescued alkaline phosphatase (AP)-positive colonies (Fig. 2B).

### NS Expression Becomes Dispensable by Forced Expression of Nanog or Esrrb in ESCs

Next, we examined whether forced expression of pluripotency marker genes shows rescue effects in *NS* tet-off ESCs, because a decline in the expression of pluripotency marker genes is one of the prominent features of these cells. Although most of the pluripotency factors did not exert any noticeable effects on the viability of *NS* tet-off ESCs (Supporting Information Fig. S2), forced expression of Nanog or Esrrb produced a number of viable and alkaline phosphatase-positive colonies (Fig. 3A). These effects were much more prominent even when compared with those obtained by simultaneous expression of Stat3 and Akt in the 2i condition. Indeed, the numbers of viable colonies were similar for Dox-treated and -untreated *NS* tet-off ESCs that had been rescued by *Nanog* or *Esrrb* expression. These rescued *NS* tet-off ESCs could be passaged and expanded without affecting their viability or the undifferentiated morphology of the colonies. Western blot analyses revealed that, unlike *NS* tet-off ESCs transfected with the empty vector, both *Nanog*- and *Esrrb*-overexpressing *NS* tet-off ESCs showed no alteration in the phosphorylation level of Stat3 after Dox-mediated ablation of *NS* expression (Fig. 3B). We also noted that the level of p53 protein was rescued by these pluripotency factors. Indeed, although obvious accumulation of p53 protein is a prominent feature of Dox-treated *NS* tet-off ESCs [1,13], p53 protein accumulation was noticeably attenuated by forced expression of constitutively active Stat3 or exposure to the 2i condition. More importantly, such accumulation became not evident by either *Nanog*- or *Esrrb*-overexpression in Dox-treated *NS* tet-off ESCs (Fig. 3B). Differences in the degrees of rescue by forced expression of either Nanog or Esrrb compared with those obtained by constitutively active Stat3 or exposure to the 2i condition were also evident for the cell proliferation rate. However, the Nanog- or Esrrb-rescued cells did not proliferate as quickly as Dox-untreated *NS* tet-off ESCs (Fig. 3C). We also confirmed that the frequency of TUNEL-positive cells with *NS* expression ablation was almost completely rescued or obviously suppressed by forced expression of Esrrb or Nanog, respectively (Fig. 3D).

### Effects of Forced Expression of Nanog and Esrrb on the Global Expression Profile of NS Expression-Ablated ESCs

**Figure 4:**
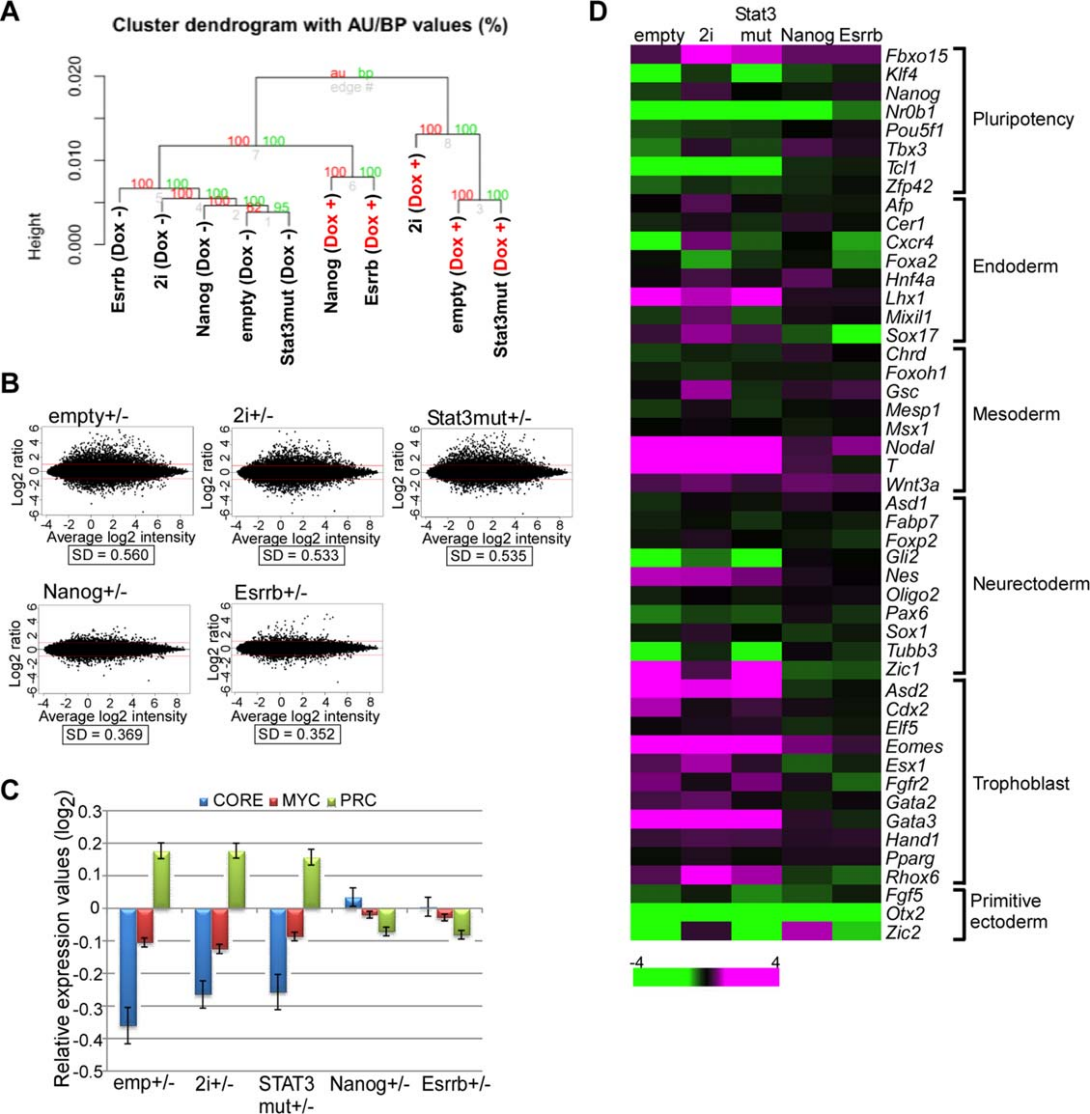
Evidence of the counteraction of nucleostemin (*NS*) expression ablation-mediated disruption of the embryonic stem cell (ESC) state by forced expression of Nanog or Esrrb from global gene expression analyses. (A): Cluster analyses of DNA microarray data from the indicated cells. The cells were cultured in the presence or absence of Dox for 6 days. (B): MA plots demonstrating a decrease in the changes of overall gene expression levels by ablation of *NS* expression in ESCs upon forced expression of Nanog or Esrrb. DNA microarray data from *NS* tet-off ESCs overexpressing either constitutively active Stat3, Nanog, or Esrrb, or exposed to the 2i condition were used to construct MA plots to compare the expression profiles in Dox-untreated and -treated conditions. *NS* tet-off ESCs transfected with an empty vector were used as controls. The SD is shown in each panel. (C): Average gene expression values of Core (blue), Myc (red), and PRC (green) module genes with respect to the indicated Dox-treated cells were calculated with standard errors using the values from their Dox-untreated counterparts as references. (D): Heat map showing expression levels of genes related to pluripotency or cellular differentiation with respect to the indicated Dox-treated cells using data from their Dox-untreated counterparts as references.

To systematically quantify the similarity in the overall gene expression profiles of *Nanog*- or *Esrrb*-rescued *NS* tet-off ESCs and Dox-untreated *NS* tet-off ESCs, we performed DNA microarray analyses and used the obtained data for cluster analyses. Data from 10 samples were clustered into two main groups in which one group mainly consisted of Dox-untreated *NS* tet-off ESCs, while three types of Dox-treated *NS* tet-off ESCs (Stat3mut, 2i, and empty) belonged to the other group. However, we noted that *Nanog*- and *Esrrb*-rescued Dox-treated *NS* tet-off ESCs showed extremely similar expression patterns. They were clearly segregated from the group of Dox-treated *NS* tet off ESCs, but belonged to the first group mainly including Dox-untreated *NS* tet-off ESCs. This result provided additional evidence that the forced expression of Nanog or Esrrb had counteracted *NS* expression ablation-mediated disruption of ESC pluripotency (Fig. 4A). MA (log ratios/mean average) plots comparing gene expression profiles (Fig. 4B) provided data consistent with this notion. The expression profile of *NS* tet-off ESCs was greatly altered upon ablation of *NS* expression with Dox and neither constitutively active Stat3 nor exposure to the 2i condition were able to alleviate this change significantly. However, forced expression of Nanog or Esrrb made *NS* tet-off ESCs rather refractory against this *NS* expression ablation-mediated change of their global expression profile. The ESC status is preserved by exquisite interplay of three transcription subnetworks termed Core, Myc, and PRC modules [31]. Our analyses of the DNA microarray data revealed that these three subnetworks were significantly unbalanced in Dox-treated *NS* tet-off ESCs, and neither expression of constitutively active Stat3 mutant nor exposure to the 2i plus LIF condition appreciably attenuated this unbalance. However, we found that these subnetworks were fairly normalized in both Nanog- and Esrrb-rescued *NS* tet-off ESCs (Fig. 4C). In addition, we used the microarray data to examine the expression levels of individual genes related to pluripotency and cellular differentiation. We found that trophoblast marker genes and pluripotency genes were significantly upregulated and downregulated, respectively (Fig. 4D). Some, but not all, mesendodermal marker genes showed elevated expression levels in Dox-treated *NS* tet-off ESCs. However, we assume that this observation did not represent the normal differentiation process of ESCs, because no clear upregulation of primitive ectodermal marker genes such as *Fgf5* and *Otx2* [32] was evident in Dox-treated *NS* tet-off ESCs at any time point. More importantly, such expression changes observed in parental *NS* tet-off ESCs were not influenced by constitutively active Stat3 or exposure to the 2i plus LIF condition, but fairly minimized by forced expression of Nanog or Esrrb. We also investigated the expression dynamics of 49 genes that have been previously denoted as Nanog-sensitive genes [33]. These genes were identified by two strict criteria: they have been demonstrated as Nanog target genes by genome-wide chromatin immunoprecipitation analyses and show immediate alterations in gene expression levels after transcriptional activation of Nanog. GSEA revealed that most genes with positively regulated expression levels by Nanog showed a decline in their expression levels after ablation of *NS* expression in ESCs, while genes subjected to negative regulation by Nanog tended to show upregulated expression levels upon *NS* expression ablation in ESCs. However, such a tendency was not observed in Nanog- or Esrrb-rescued *NS* tet-off ESCs (Supporting Information Fig. S3). It may be noteworthy that Nanog-rescued *NS* tet-off ESCs tended to show relatively higher expression of genes subjected to positive regulation by Nanog when the cells were treated with Dox compared with that in Dox-untreated cells. These data imply that abnormally high levels of Nanog in Nanog-overexpressing Dox-untreated cells may be detrimental for the expression of Nanog target genes.

**Figure 5:**
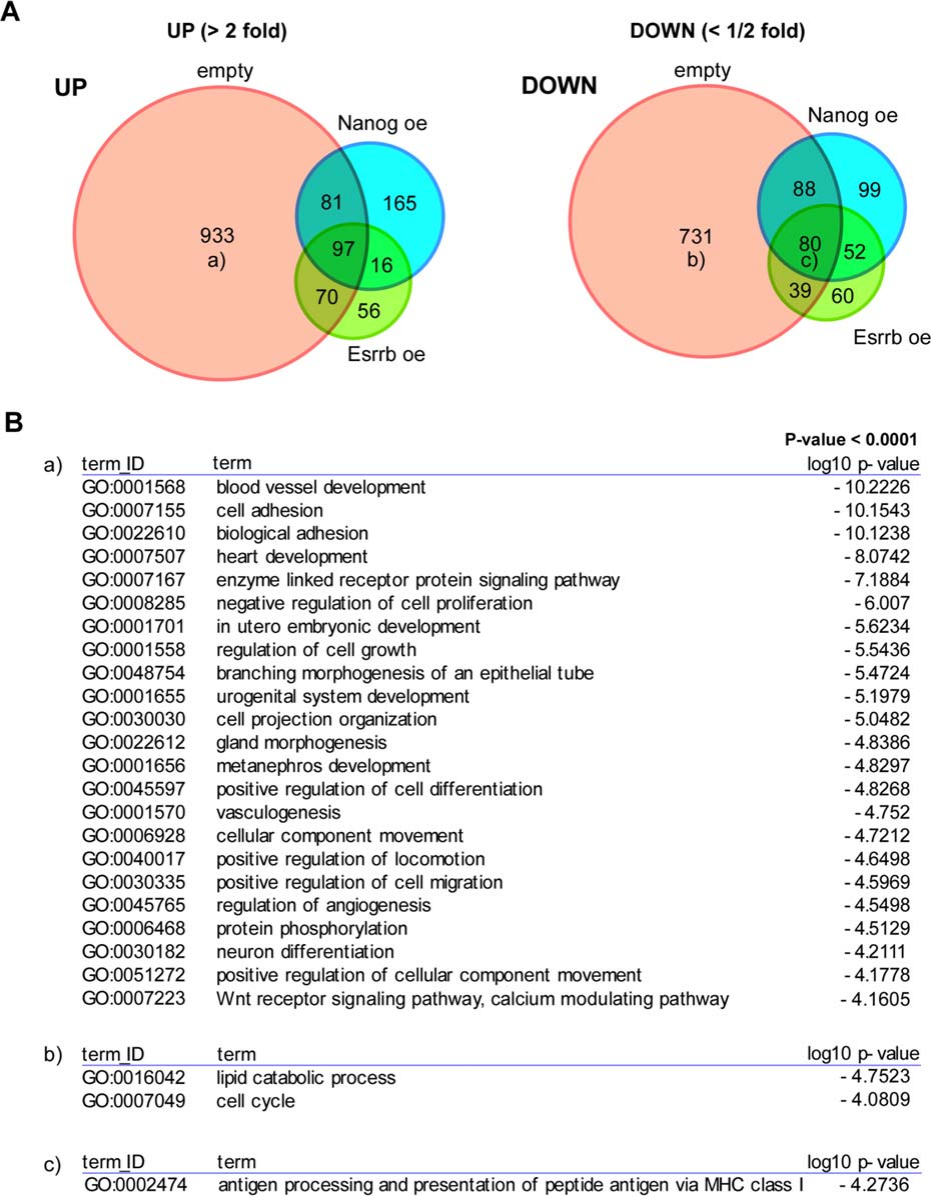
Characterization of the effects of forced expression of Nanog or Esrrb on the global expression profile of nucleostemin expression-ablated embryonic stem cells (ESCs). (A): Venn diagram illustrating relative relationships of upregulated (left) and downregulated (right) genes in Dox-treated ESCs with stable integration of either Nanog (blue), Esrrb (green,) or empty (red) expression vectors. Small letters (a, b, and c) in circles represent groups of gene sets that were suggested to be enriched with certain biologically related genes by the gene ontology (GO) analyses shown in (B). (B): GO analyses of the gene sets depicted in (A) using DAVID. The *p*-value cutoff was set to <1 × 10^−4^. All gene sets (seven groups in each panel) were subjected to the analyses. However, prominent GO terms that satisfied the criteria of selection (*p* < 1 × 10^−4^) were only identified for the three gene sets denoted with a, b, and c in (A).

### GO Analyses of Genes Showing Differential Expression Levels by Ablation of NS Expression in ESCs

Next, to correlate upregulation and downregulation of these genes by *NS* expression ablation with overall molecular functions, we conducted GO analyses. To this end, we first constructed Venn diagrams with genes that showed altered expression levels by more than twofold in Dox-treated *NS* tet-off ESCs that were transfected with either *Nanog*, *Esrrb*, or an empty vector (Fig. 5A). We found that the expression levels of most genes (933 out of 1,181) that were upregulated by more than twofold in Dox-treated *NS* tet-off ESCs were normalized by forced expression of Nanog or Esrrb. Similarly, 731 out of 938 genes that were downregulated in Dox-treated *NS* tet-off ESCs were normalized by Nanog or Esrrb expression. Next, we conducted GO classification analyses. A number of GO terms, especially those related to morphogenesis and development, were significantly enriched for genes upregulated but normalized by Nanog or Esrrb (Fig. 5Ba), while only two GO terms (“lipid catabolic process” and “cell cycle”) were represented as prominent terms among genes downregulated but normalized by Nanog or Esrrb (Fig. 5Bb). These results indicate that the major role of NS in ESCs is suppression of cellular differentiation and morphogenesis, which can be substituted by forced expression of either Nanog or Esrrb. These data also indicated that, unlike the role of upregulated genes, the downregulated genes are a gene set without an apparent overall biological significance. We also subjected other groups of genes in the Venn diagrams shown in Figure 5A to GO analyses. However, none of the GO terms were selected by the analyses except for genes with downregulated expression in *NS* tet-off ESCs irrespective of the forced expression of either Nanog or Esrrb. In this case, genes categorized into a GO term (antigen processing and presentation of peptide antigen via MHC class I) were found to be enriched, but the physiological significance of identification of this term is unknown at present (Fig. 5Bc).

### Evidence of a NS-Mediated Rad51-Independent Mechanism to Preserve Cell Viability and Expression of Pluripotency Marker Genes in ESCs

**Figure 6:**
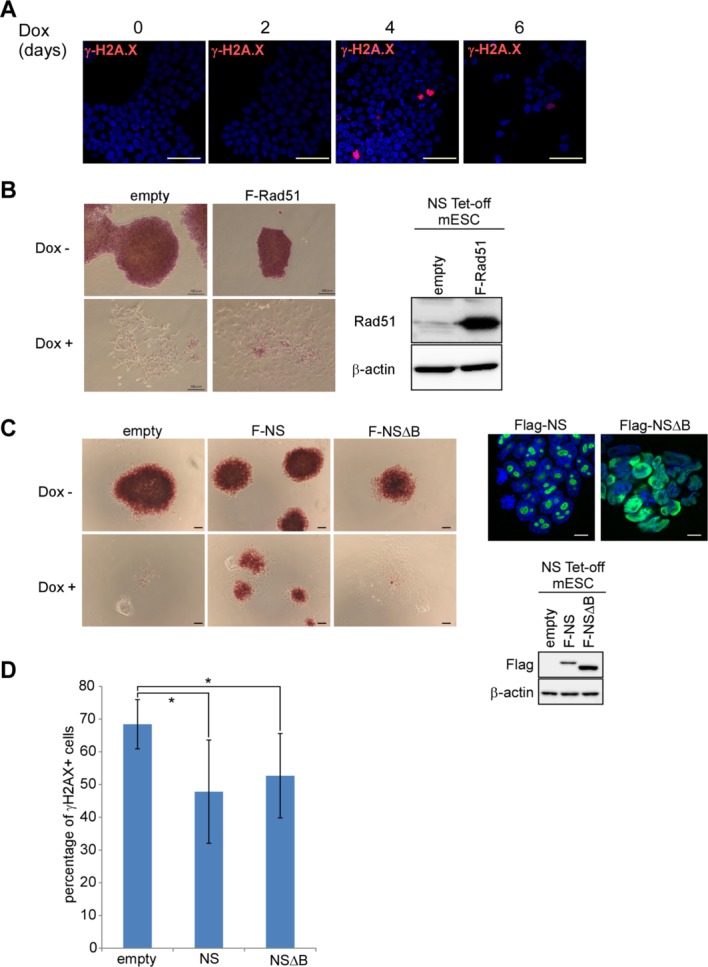
Evidence of an additional role of NS other than a genome-protective role in ESCs. (A): Examination of γ-H2A.X signals in *NS* tet-off ESCs. *NS* tet-off ESCs were cultured with Dox for 0, 2, 4, and 6 days, and then immunostained with an anti-γ-H2A.X antibody. Scale bar = 10 µm. (B): Alkaline phosphatase staining of control and Flag-Rad51-overexpressing *NS* tet-off *ESCs* cultured in the absence or presence of Dox for 6 days. Scale bar = 100 µm (left panel). Western blot analyses were also performed to confirm the exogenously overexpressed Flag-tagged Rad51 with an antibody against Rad51 (right panel). (C): Alkaline phosphatase staining of control, Flag-NS-, and Flag-NSΔB-overexpressing *NS* tet-off ESCs that were cultured either with or without Dox for 5 days. Scale bar = 100 µm (left panel). Immunostaining of these cells with an anti-Flag antibody was performed to confirm nucleolar and nuclear localization of wild-type NS and the NSΔB mutant, respectively. Scale bar = 10 µm (right upper panel). Right lower panel shows a Western blot of exogenously overexpressed wild-type NS and the NSΔB mutant. (D): Counteracting effect of overexpression of wild-type NS and the NSΔB mutant on HU-mediated genomic damage in ESCs. Levels of genomic DNA damage were evaluated by positivity for γ-H2A.X in immunocytochemical analyses. *, *p* < .05. Abbreviations: ESC, embryonic stem cell; NS, nucleostemin.

Recently, it has been demonstrated that recruitment of Rad51 to foci of stalled replication-induced DNA damage to maintain genomic stability is the major role of NS in NSCs [6]. To investigate whether the same mechanism underlies the apoptotic phenotypes of *NS* tet-off ESCs, we first examined whether there was a striking increase of γ-H2AX-positive cells among *NS* tet-off ESCs, which is observed with *NS*-null NSCs. We found no evident increase of γ-H2AX-positive cells for the first 2 days after Dox addition. We did observe γ-H2AX-positive *NS* tet-off ESCs at 4 days post-Dox administration. However, these cells never showed dot-like signal pattern, but homogeneously stained pattern in the nucleus, implying that double-stranded DNA breaks marked by γ-H2AX positivity were not the primary cause, but rather a secondary consequence of apoptotic cell death of *NS* tet-off ESCs (Fig. 6A) [34,35]. Differences in the molecular mechanisms of *NS* tet-off ESC and NSC apoptosis were also suggested by the fact that, unlike *NS* knockout NSCs, overexpression of neither Rad51 (Fig. 6B) nor the nucleolar localization-defective NS mutant (NSΔB) (Fig. 6C) mitigated the detrimental phenotypes of *NS* tet-off ESCs. Thus, these results indicate that loss of the stabilizing effect of NS on the genome does not account for the major cause of the extensive cell death phenotypes of *NS* tet-off ESCs. However, these results do not necessarily eliminate the possibility that NS also participates in genomic stabilization of ESCs. NS protein in ESCs may exert dual functions to preserve pluripotency and cell viability by preservation of genome integrity, as demonstrated in other *NS*-expressing cells such as NSCs, and some ESC-specific function. To test this hypothesis, we overexpressed NS in wild-type ESCs and compared the levels of γ-H2AX positivity after induction of DNA damage by HU treatment with those in ESCs transfected with an empty vector. These analyses revealed that the numbers of γ-H2AX-positive cells were noticeable declined by *NS* overexpression and this effect was evident even with NSΔB (Fig. 6D). Taken together, these results indicate that the genome-protective role of NS is operative at least among ESCs, NSCs, and regenerating hepatocytes, and probably more widely among *NS*-expressing cells. Our data also indicate that NS in ESCs is involved in an additional mechanism in parallel to preserve pluripotency and cell viability.

### Crucial Role of NS in Preservation of Pluripotency Marker Gene Expression and Cell Viability of EpiSCs

**Figure 7:**
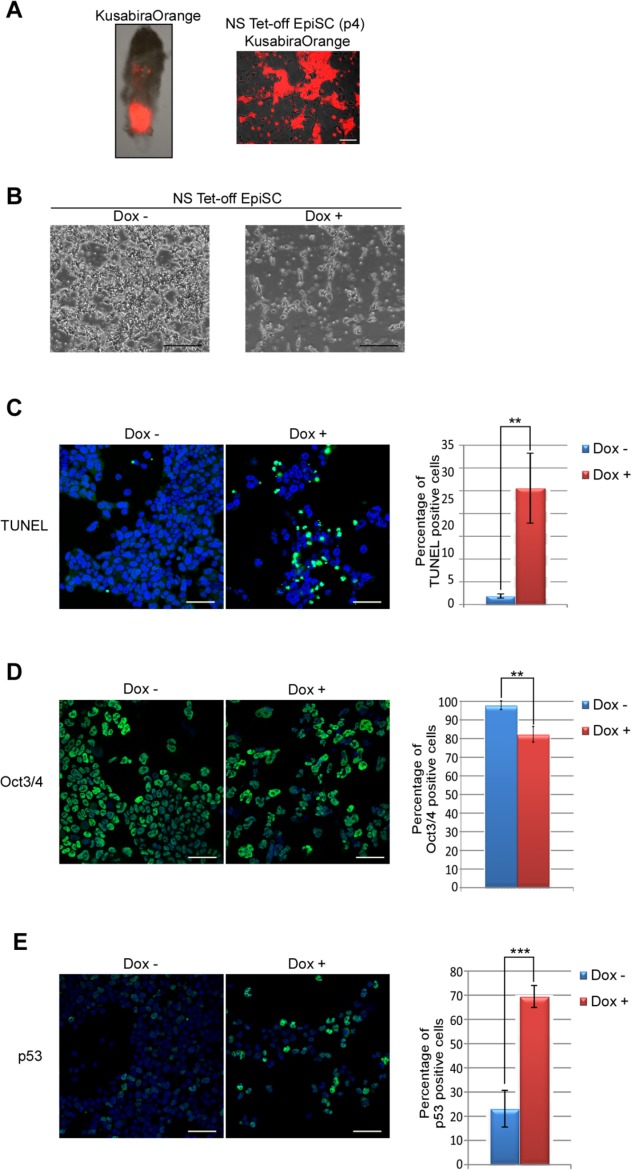
*NS* expression is indispensable for cell viability and preservation of pluripotency marker expression in EpiSCs. (A): A 6.5 dpc embryo that was injected with fluorescent Kusabira Orange (KBO)-overexpressing *NS* tet-off embryonic stem cells (ESCs) at the blastocyst stage (left panel). *NS* tet-off EpiSCs from the 6.5 dpc embryo bearing cells derived from KBO-marked *NS* tet-off ESCs (right panel). Scale bar = 100 µm. Puromycin selection was applied to eliminate recipient cell-derived EpiSCs. (B): Microscopic inspection of *NS* tet-off EpiSCs that were untreated or treated with Dox for 5 days. Scale bar = 500 µm. **, *p* < .01. (C): TUNEL assays of *NS* tet-off EpiSCs that were untreated or treated with Dox for 6 days. The ratio of TUNEL-positive cells is shown as the mean with SD. Scale bar = 50 µm. **, *p* < .01. (D): Oct3/4 signals in *NS* tet-off EpiSCs that were untreated or treated with Dox for 6 days. The ratio of Oct3/4-positive cells is shown as the mean with SD. Scale bar = 50 µm. **, *p* < .01. (E): p53 signals in *NS* tet-off EpiSCs that were untreated or treated with Dox for 6 days. The ratio of p53-positive cells is shown as the mean with SD. Scale bar = 50 µm. ***, *p* < .001. Abbreviations: EpiSC, epiblast stem cell; NS, nucleostemin.

Both ESCs and EpiSCs exhibit pluripotency [14,15]. Therefore, we determined whether NS also participates in preserving the pluripotency of EpiSCs. To test this hypothesis directly, we generated *NS* tet-off EpiSCs (Fig. 7A) and then cultured these cells with or without Dox. Microscopic observation revealed that Dox-treated *NS* tet-off EpiSCs showed severely impaired cell growth (Fig. 7B). Similar to ESCs, this severe phenotype was not alleviated by forced expression of Rad51 (Supporting Information Fig. S4). A substantial number of cells also showed positivity for TUNEL signals (Fig. 7C). Furthermore, our immunocytochemical analyses revealed that substantial proportions of Dox-treated *NS* tet-off EpiSCs became negative for Oct-3/4 (Fig. 7D), indicating cellular differentiation. Because accumulation of p53 protein is one of the prominent features of *NS* tet-off ESCs, we examined whether p53 protein was similarly accumulated in *NS* tet-off EpiSCs. Our analyses revealed that a substantial number of cells expressed p53 protein in their nucleus after Dox-mediated ablation of *NS* expression (Fig. 7E). Next, we examined the consequence of *Nanog* or *Esrrb* overexpression in *NS* tet-off EpiSCs. These analyses revealed that, unlike *NS* tet-off ESCs, forced expression of neither Nanog nor Esrrb overrode the requirement of NS for the preservation of robust cell proliferation, cell viability, and homogeneous expression of Oct3/4 in EpiSCs (Supporting Information Fig. S5). Similar to ESCs, these results indicate that NS crucially participates in preservation of the cell viability of EpiSCs. However, there is a definite difference in the responses to forced expression of either Nanog or Esrrb in *NS* tet-off ESCs and EpiSCs. We will discuss about these data more in details later (see Discussion).

### Role of NS Expression in Human iPSCs

To extend our findings concerning the role of *NS* expression in mouse ESCs and EpiSCs, we examined the role of *NS* expression in the preservation of human iPSC viability by knockdown experiments. We found that a proportion of human iPSCs bearing activated caspase-3/7 became significantly larger as a result of shRNA-mediated knockdown of *NS* expression compared with control cells in which shRNA-mediated knockdown targeted unexpressed luciferase (Luc) (Supporting Information Fig. S6). This result implies that *NS* also plays crucial roles in preserving the viability of human iPSCs.

## Discussion

NS is involved in numerous biological functions, and expressed in various stem/progenitor cell types, including ESCs. Knockout mouse analyses have demonstrated overt morphological abnormalities in *NS* knockout embryos during blastocyst formation, in which a discernible inner cell mass is absent at 3.5 dpc and the overall structure of the embryo resembles morulae without a blastocoel cavity [3]. In accordance with these findings, we have previously demonstrated that *NS* expression is essential to preserve the cell viability of ESCs that are derivatives of the inner cell mass of blastocysts [13]. Subsequently, Qu and Bishop [4] have demonstrated that *NS* expression is important to preserve the unique cell cycle kinetics observed in mouse ESCs that show an unusually low proportion of cells in G1 phase. One of the major findings in this study is that forced expression of Nanog or Esrrb rendered *NS* expression in ESCs redundant. Indeed, Nanog- or Esrrb-overexpressing *NS* tet-off ESCs were able to maintain normal expression levels of pluripotency marker genes and propagate robustly for prolonged periods without showing any apparent apoptotic phenotype. Strong counteracting effects of Nanog and Esrrb against the changes associated with *NS* expression ablation in ESCs were also suggested by analyses of the global gene expression profile. Approximately 80% of genes with altered expression levels by more than twofold were not strongly affected by the loss of *NS* expression in *Nanog*- or *Esrrb*-overexpressing ESCs. GO analyses revealed that a number of terms related to morphogenesis and development were significantly represented among genes upregulated by more than twofold in *NS* expression-ablated ESCs, but became insensitive to the loss of *NS* expression by forced expression of *Nanog* or *Esrrb*. The same analyses of downregulated genes but normalized by Nanog or Esrrb revealed only two GO terms (lipid catabolic and cell cycle). Therefore, these results indicate that the major functions of NS in ESCs involve suppression of cellular differentiation and promotion of robust cell proliferation, which can be replaced by Nanog or Esrrb. Recently, Esrrb has been shown to be one of the major targets of Nanog [33]. Indeed, Esrrb can functionally substitute Nanog in ESCs. Therefore, it is possible that the rescue effect of *Nanog* overexpression may be largely explained by its function in supporting *Esrrb* gene expression.

It has been recently demonstrated that NS protein is involved in maintaining genomic stability of NSCs and regenerating liver cells by facilitating recruitment of Rad51 to foci of stalled DNA replication-induced DNA damage [6,7]. However, our data obtained by forced expression of Rad51 and other experimental data demonstrate that impairment of this mechanism at least does not account for the major causes of the detrimental phenotypes observed in *NS* tet-off ESCs and EpiSCs. These data imply the presence of a pluripotent cell-specific function of NS protein. Similar to ESCs, our data also showed that *NS* expression is crucial in EpiSCs to preserve their pluripotency, self-renewal, and cell viability. However, unlike in ESCs, no apparent rescue effect by forced expression of Nanog or Esrrb was evident in *NS*-null EpiSCs. The endogenous expression levels of *Nanog* and particularly *Esrrb* were significantly low in EpiSCs compared with those in ESCs [36]. Therefore, we assume that these differential counteracting effects by forced expression of Nanog or Esrrb against *NS* expression ablation in ESCs and EpiSCs are due to the lack of additional factors and/or cofactors in EpiSCs, which are required for the effectiveness of the forced expression of these factors.

Our data demonstrated that overexpression of NS mitigated the DNA damage caused by addition of HU, suggesting that the genome-protecting role of NS in NSCs and regenerating hepatocytes is also operative in ESCs. However, our data demonstrated that an additional mechanism is operating in parallel to preserve pluripotency and cell viability, although the molecular basis of such a role is unknown at present except that it can be suppressed by forced expression of Nanog or Esrrb. Our finding of the partial rescue of *NS* tet-off ESCs by Stat3, Akt, and the 2i condition may help to elucidate the pluripotent cell-specific role of NS. Prominent stabilization of p53 in *NS* tet-off ESCs may be an alternative, but not mutually exclusive, clue to understand the molecular basis of NS function in pluripotent cells, because p53 is known to repress the expression of numerous pluripotency maker genes including Nanog [37]. It is also important to determine whether the pluripotent cell-specific role of NS we advocated in this study is truly specific in pluripotent cells or operates in other cell types. Significant similarity has been demonstrated in the gene expression profiles of pluripotent cells and cancer cells [31,38]. Therefore, it is tempting to speculate that NS participates in preserving the undifferentiated state and cell viability of cancer cells, particularly cancer stem cells, in a similar manner that operates in ESCs and EpiSCs. This hypothesis can be tested experimentally, because cancer cells such as glioma cells can be theoretically generated from ESC-derived NSCs/progenitor cells [39,40].

## Conclusions

Previous knockdown and knockout studies have demonstrated that NS is important to preserve the self-renewal and high expression levels of pluripotency marker genes in ESCs. In this study, we demonstrate that *NS* expression is also essential to preserve the self-renewal and pluripotent properties of EpiSCs. Furthermore, our data demonstrate that forced expression of Nanog or Esrrb renders *NS* expression dispensable in ESCs, but not EpiSCs.
