# Supplementary figures and images for: Forced Expression of Nanog or Esrrb Preserves the ESC Status in the Absence of Nucleostemin Expression
Source: Stem Cells. 2014 Dec 18;33(4):1089–101. doi: 10.1002/stem.1918 (PMC4409032; doi:10.1002/stem.1918)

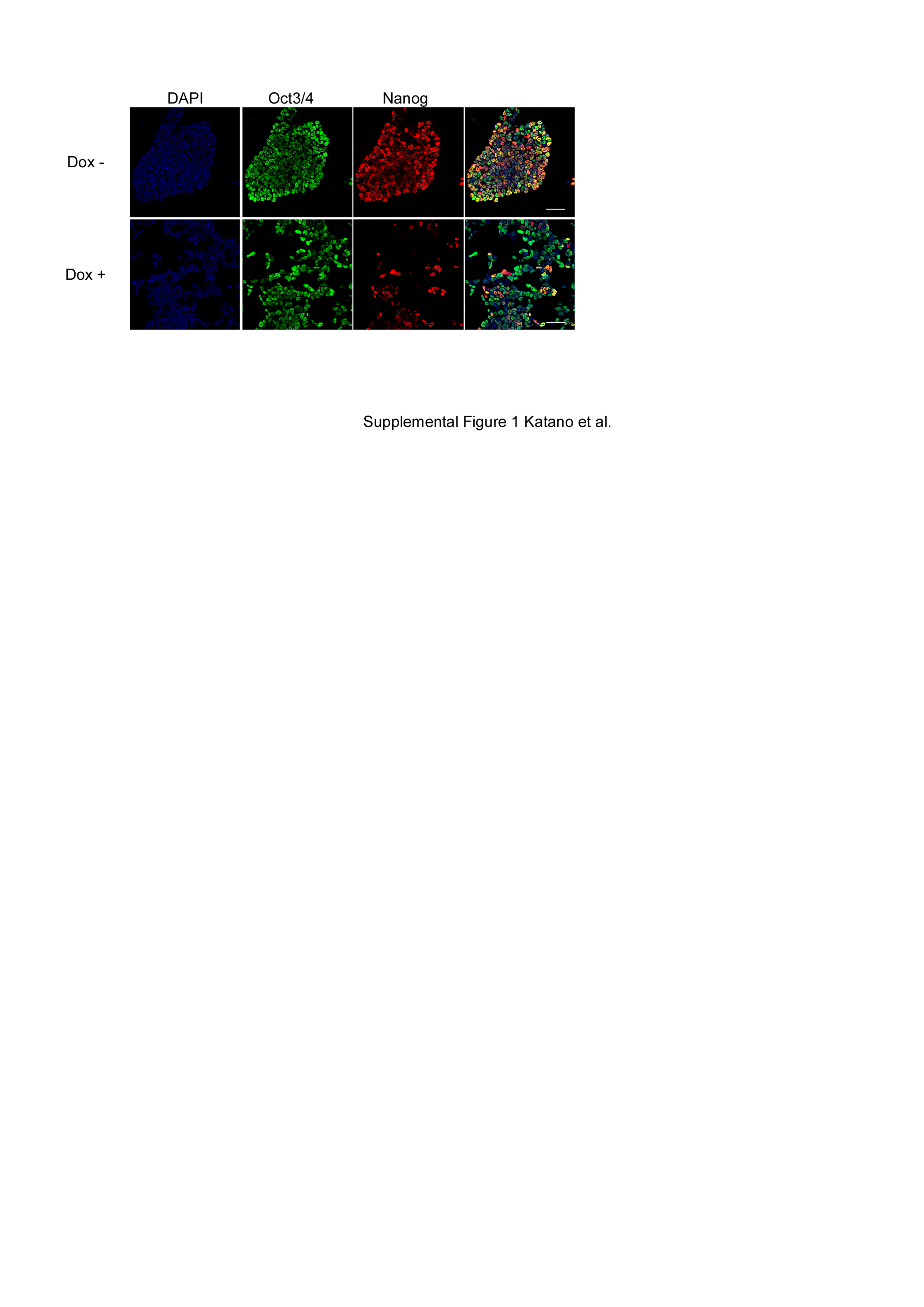

Supplement: Supplementary file 1 — Supplementary Figure 1 [file stem0033-1089-sd1.tif]

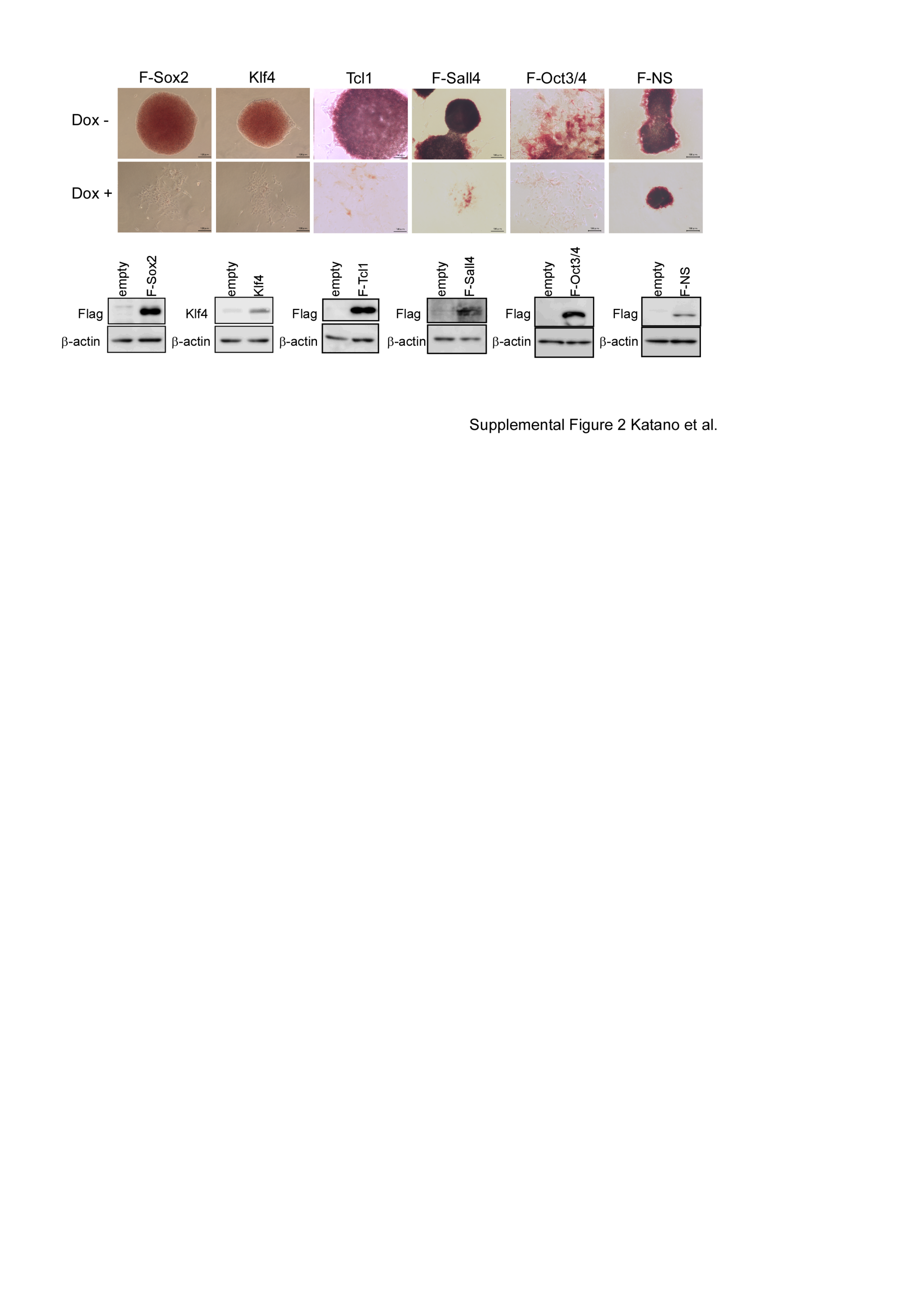

Supplement: Supplementary file 2 — Supplementary Figure 2 [file stem0033-1089-sd2.tif]

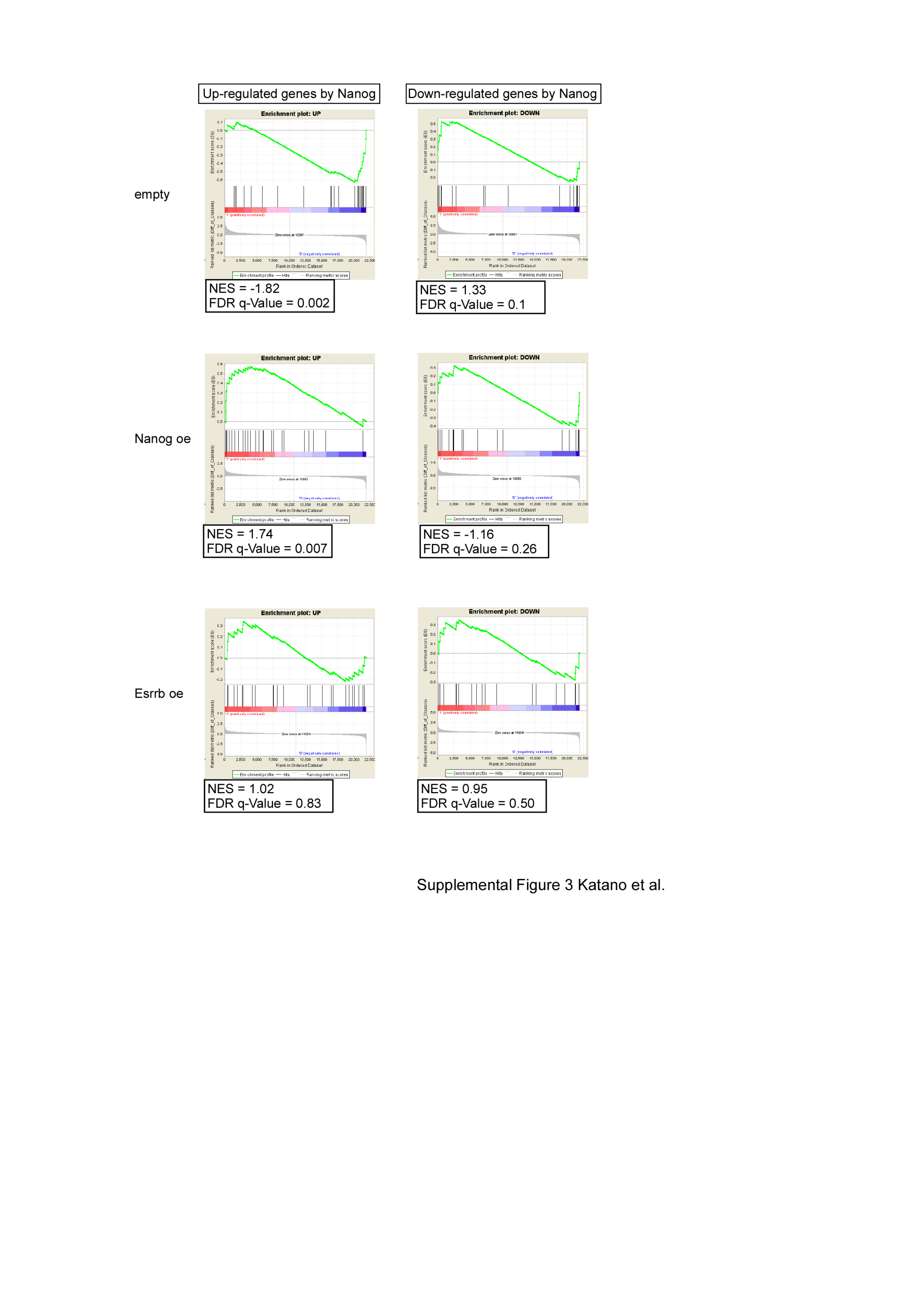

Supplement: Supplementary file 3 — Supplementary Figure 3 [file stem0033-1089-sd3.tif]

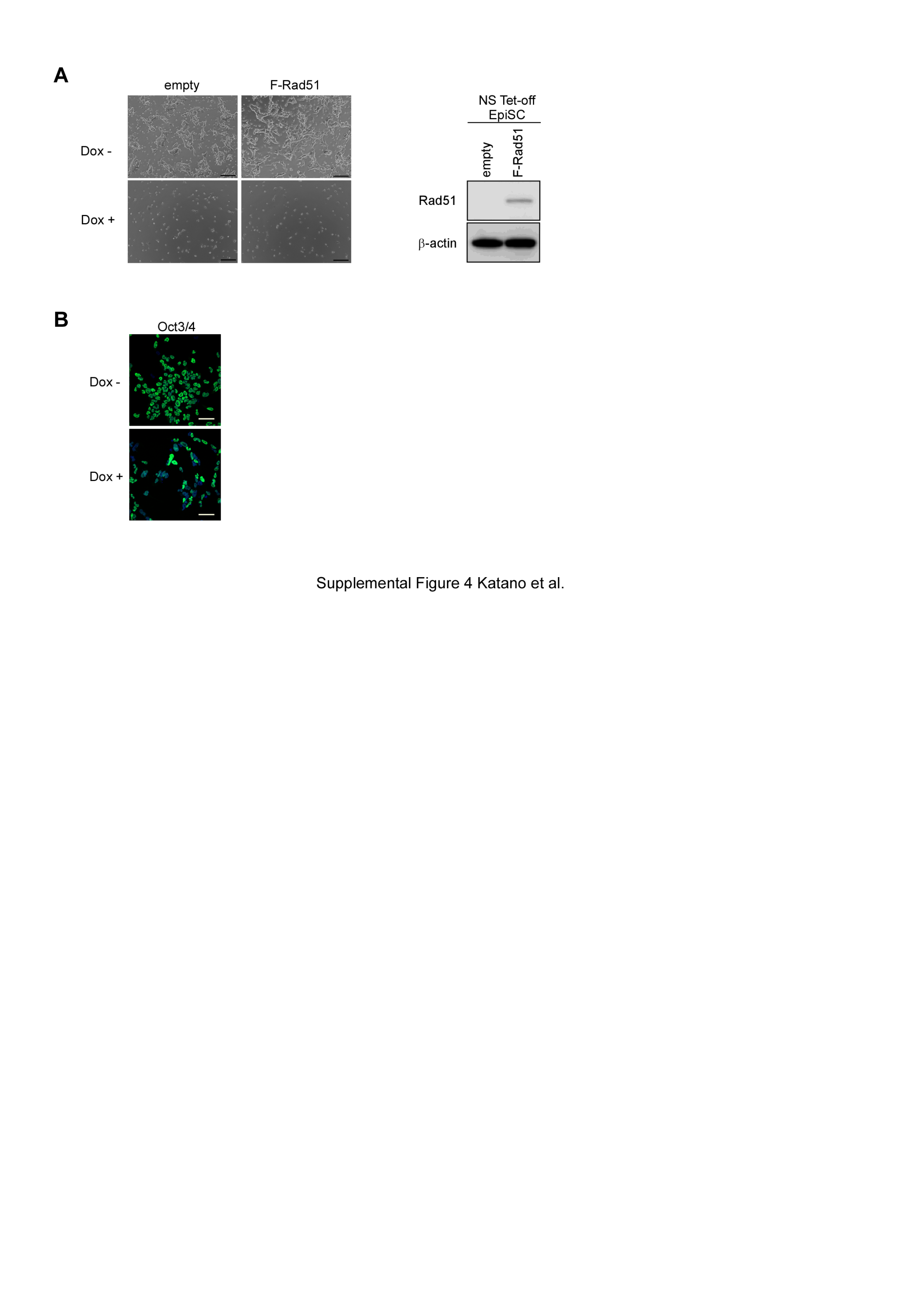

Supplement: Supplementary file 4 — Supplementary Figure 4 [file stem0033-1089-sd4.tif]

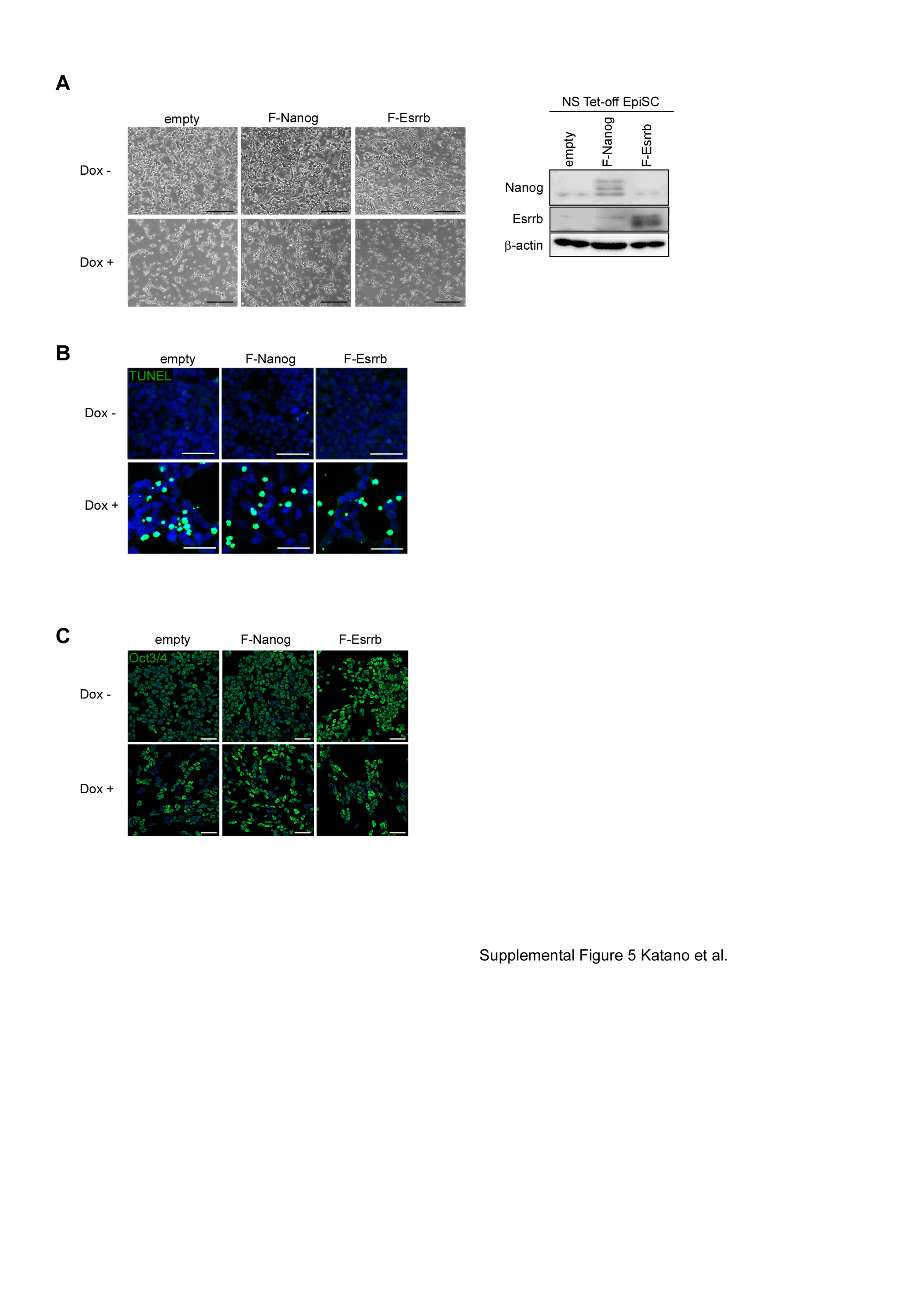

Supplement: Supplementary file 5 — Supplementary Figure 5 [file stem0033-1089-sd5.tif]

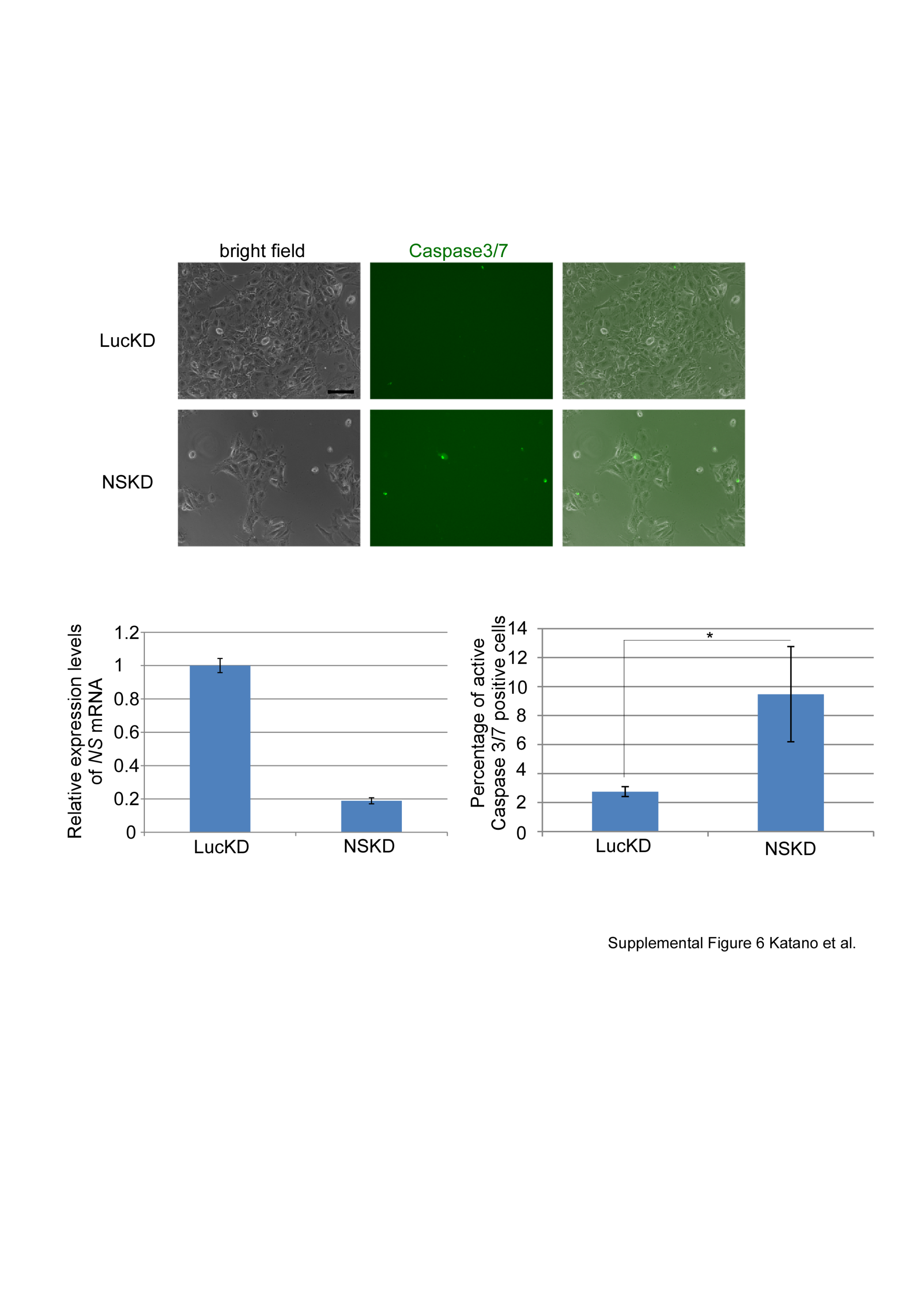

Supplement: Supplementary file 6 — Supplementary Figure 6 [file stem0033-1089-sd6.tif]
