# Supplementary material for: Forced Expression of Nanog or Esrrb Preserves the ESC Status in the Absence of Nucleostemin Expression
Source: Stem Cells. 2014 Dec 18;33(4):1089–101. doi: 10.1002/stem.1918 (PMC4409032; doi:10.1002/stem.1918)
Supplement: Supplementary file 7 — Supplementary Materials and Methods [file stem0033-1089-sd7.docx]

**Supplemental materials and methods**

**Reagent and antibodies**

The following reagents were used at the indicated concentrations: 3 μM GSK-3 inhibitor CHIR99021 (Axon Medchem, Groningen, Netherlands, <http://www.axonmedchem.com>); 1 μM MEK inhibitor PD0325901 (Axon Medchem); 1 μg/mL Dox (631311) (Clontech, Mountain View, CA, http://www.clontech.com); anti-NS (AB5691), anti-Sox2 (Ab5603), anti-Prdm14 (AB4350) (Millipore, Billerica, MA, <http://www.millipore.com>); anti-Klf4 (AF3158) (R&D Systems); anti-Nanog (RCAB0001P) (Cosmo Bio, Tokyo, Japan, <http://www.cosmobio.co.jp>); anti-GSK3β (610202) (BD Biosciences, San Jose, CA, http://www.bdbiosciences.com); anti-Akt (#9272), anti-phospho-Akt (Ser473) (#9271), anti-STAT3 (124H6) (#9139), anti-phospho-STAT3 (Tyr705) (D3A7) (#9145), anti-p44/42 (ERK1/2) (#9102), anti-phospho-p44/42 (ERK1/2) (Thr202/Tyr204) (#9101), anti-phospho-GSK3β (Ser9) (#9336), anti-phospho-histone H2A.X (Ser139) (20E3) (#9718), anti-p53 (1C12) (#2524) (Cell Signaling Technology, Danvers, MA, <http://www.cellsignal.com>); anti-Oct3/4 (C-10) (sc-5279), anti-β-actin (C4) (sc-47778) (Santa Cruz Biotechnology, Dallas, Texas, <http://www.scbt.com>); anti-Esrrb (PP-H6705-00) (PPMX, Tokyo, Japan, <http://www.ppmx.com>); anti-p53 (NCL-p53-CL5P) (400511) (Novocastra, Newcastle upon Tyne, UK, http://www.novocastra.co.uk).

**Vector construction**

Vectors used for rescue experiments of *NS* tet-off ESCs and/or EpiSCs were as follows: pCAG-Flag-Nanog-IresPuro(IP), pCAG-Flag-Nanog-IresHygro(IH), pCAG-Flag-Esrrb-IP, pCAG-Flag-Esrrb-IH, pCAG-Flag-NS-IP, pCAG-NS-IP, pCAG-NSΔB-IP, pCAG-Flag-STAT3mut-IP, pCAG-Flag-Rad51-IP, pCAG-Flag-Oct3/4-IP, pCAG-Flag-Sox2-IP, pCAG-Klf4-IP, pCAG-Flag-Sall4-IP, and pCAG-Flag-Tcl1-IP. The plasmids were constructed by subcloning cDNAs into the Xho1/Not1 sites of either pCAG-IP or pCAG-IH in which the puromycin resistance gene of pCAG-IP had been replaced with a hygromycin resistance gene. The vectors were transfected into *NS* Tet-off ESCs by Lipofectamine 2000 (Life Technologies, 11668019). Transfection of vectors into EpiSCs was conducted with a Nucleofector™ 2b (AAB-1001) (LONZA, Basel, Switzerland, http://www.lonza.com) using a Mouse ES Cell Nucleofector Kit (VPH-1001) and the A-024 program.

**Western blotting and immunostaining**

For western blotting, cellular proteins were recovered by treatment with a lysis buffer (50 mM Tris-HCl [pH 7.5], 150 mM NaCl, 0.5% Triton X-100, and 1 mM EDTA) containing 1× Halt Phosphatase Inhibitor (#78420) (Thermo Scientific, Waltham, MA, <http://www.thermoscientific.com>). The proteins were resolved by sodium dodecyl sulfate-polyacrylamide gel electrophoresis, transferred to a polyvinylidene difluoride membrane, and probed by the indicated primary antibodies and appropriate secondary antibodies conjugated to horseradish peroxidase. Specific protein bands were detected by ECL Western Blotting Detection Reagents (GE Healthcare, Piscataway, NJ, <http://www.gehealthcare.com>). For immunocytochemistry, cells were plated on 0.1% gelatin-coated Cell Disks (MS-92132) (SUMITOMO BAKELITE Co., Tokyo, Japan, <http://www.sumibe.co.jp/english>) and then fixed with 4% paraformaldehyde for 20 min at room temperature. After three washes with phosphate buffered saline (PBS), the cells were permeabilized and blocked in blocking buffer (3% bovine serum albumin (BSA), 0.3% Triton X-100, and 1% heat-inactivated normal sheep serum in PBS) for 30 min at room temperature. After washing with PBS, primary antibodies in blocking buffer were added, followed by incubation at 4°C for overnight. After three washes with PBS, the cells were incubated with the appropriate Alexa Fluor dye-conjugated secondary antibodies (Invitrogen, Carlsbad, CA, http://www.lifetechnologies.com) and 3 μg/mL DAPI in PBS containing 1% BSA at 37°C for 1 h. The cells were then washed and observed under a confocal laser scanning microscope.
